# Efforts towards overcoming drought stress in crops: Revisiting the mechanisms employed by plant growth-promoting bacteria

**DOI:** 10.3389/fmicb.2022.962427

**Published:** 2022-07-29

**Authors:** Ayomide Emmanuel Fadiji, Gustavo Santoyo, Ajar Nath Yadav, Olubukola Oluranti Babalola

**Affiliations:** ^1^Food Security and Safety Focus Area, Faculty of Natural and Agricultural Sciences, North-West University, Mmabatho, South Africa; ^2^Instituto de Investigaciones Químico Biológicas, Universidad Michoacana de San Nicolás de Hidalgo, Morelia, Mexico; ^3^Microbial Biotechnology Laboratory, Department of Biotechnology, Eternal University, Baru Sahib, India

**Keywords:** drought, food production, phytohormones, plant growth promotion, sustainability

## Abstract

Globally, agriculture is under a lot of pressure due to rising population and corresponding increases in food demand. However, several variables, including improper mechanization, limited arable land, and the presence of several biotic and abiotic pressures, continually impact agricultural productivity. Drought is a notable destructive abiotic stress and may be the most serious challenge confronting sustainable agriculture, resulting in a significant crop output deficiency. Numerous morphological and physiological changes occur in plants as a result of drought stress. Hence, there is a need to create mitigation techniques since these changes might permanently harm the plant. Current methods used to reduce the effects of drought stress include the use of film farming, super-absorbent hydrogels, nanoparticles, biochar, and drought-resistant plant cultivars. However, most of these activities are money and labor-intensive, which offer limited plant improvement. The use of plant-growth-promoting bacteria (PGPB) has proven to be a preferred method that offers several indirect and direct advantages in drought mitigation. PGPB are critical biological elements which have favorable impacts on plants’ biochemical and physiological features, leading to improved sugar production, relative water content, leaf number, ascorbic acid levels, and photosynthetic pigment quantities. This present review revisited the impacts of PGPB in ameliorating the detrimental effects of drought stress on plants, explored the mechanism of action employed, as well as the major challenges encountered in their application for plant growth and development.

## Introduction

Among the main abiotic pressures endangering food security, drought is one of the most damaging and catastrophic. Drought stress has detrimental consequences for social, economic, and environmental systems, including forests, shrubs, and grasslands. Recent findings suggest droughts have serious impacts on the pools, processes and fluxes of terrestrial carbon and nitrogen cycles in these three ecosystem types (Deng et al., 2021). Drought is also expected to affect plant growth and agricultural output in more than half of arable areas by 2050 under the present climate change scenario (Kasim et al., 2013). Therefore, drought is a complex abiotic stress that affects plants from the to the molecular levels, resulting in yield loss (Wang X. et al., 2016; Kaur and Asthir, 2017). Its effects on food security are, however, being addressed globally *via* comprehensive measures (Grover et al., 2011).

Farmers deal with drought as a serious issue yearly. Drought is a natural occurrence that is brought on and sustained over an extended period by a lack of fresh water supply to meet human and ecological demands (Balint et al., 2013). Monitoring drought is often difficult as it is typically not confined to a particular time frame or an area (Salehi-Lisar and Bakhshayeshan-Agdam, 2016). This multifaceted stress mostly emanates from declining rainfall and a subsequent dry spell. Drought stress is divided into four different types: hydrological, meteorological, socio-economic, and agricultural drought (Ahluwalia et al., 2021). Hydrological drought takes place in locations with a scarce and limited supply of water supply, notably on the ground and surface levels. Meteorological drought takes place in locations with dry weather; socio-economic drought takes place in situations where there is an intense scarce and low supply of water; agricultural drought is often linked to a reduction in the level of soil water levels and consequent crop failures, majorly affecting the global food production (Heim and Richard, 2002; Ahluwalia et al., 2021).

Aside the fact that it affects global food production, drought also causes water quality deterioration, worsens soil erosion, and disasters such as floods, fires, and the spread of diseases. According to the 2018 report of the United Nations World Water Development, an estimated 55 million people are impacted by drought globally, and 700 million people might be displaced because of it by 2030 (WWAP, 2018). The socio-economic effects of drought also result in significant financial losses. For instance, the prolonged California drought resulted in around 3.8 billion dollars in agricultural losses, with 1.7 billion dollars in crop income losses between 2014 and 2016 (Howitt et al., 2015). Similar to this, the 2005 Spain’s Ebro River Basin drought had an estimated economic of about 0.57 billion dollars (Juana et al., 2014).

Interestingly, cultivars that can withstand drought stress, genetic engineering procedures, crop calendar adjustments, and resource management practices have all been studied extensively across the world as potential solutions to alleviate moisture stress, however, a number of these techniques are onerous (Philippot et al., 2013). Nevertheless, these methods are costly, stressful, consume more time and may result in the loss of the desired feature in the host’s gene pool (Philippot et al., 2013). Furthermore, under the terms and conditions of national rules, genetically altered plants are not readily approved (Kasim et al., 2013). In this way, microbes are an underappreciated contributor to increasing plant drought resistance (Kang et al., 2014b; Singh et al., 2016). As a result, a focus on sustainable agriculture, food security, and protection of the environment improves when utilizing the potential of beneficial microorganisms, such as plant growth-promoting bacteria or PGPB (Ilangumaran and Smith, 2017). Screening and selection of drought stress tolerance PGPB and its usage in plants, according to Kaushal and Wani (2016), might assist to overcome productivity barriers in drylands.

Furthermore, PGPB, which are natural soil inhabitants of the rhizosphere as well as excellent colonizers of plant internal compartments and surfaces, may efficiently overcome the harmful effects of drought stress in plants (Sati et al., 2022). *Azospirillum*, *Azotobacter*, *Bacillus*, *Klebsiella*, *Paenibacillus*, *Pseudomonas*, *Rhizobium*, and *Serratia*, are the popular plant-associated PGPB genera (Abdelaal et al., 2021). Of course, other PGPB genera have shown beneficial abilities to ameliorate drought stress in plants (Manjunatha et al., 2022). There are novel and new strains, which are emerging with new and broad potential as bioinoculants, showing that most of these bacteria can improve growth characteristics and yield parameters in most crops exposed to different abiotic stresses under natural circumstances through the synthesis of phytohormones, amino acids, enhancement of nutrient availability and nitrogen fixation (Alkahtani et al., 2020a,b).

Through the production of indole acetic acid (IAA), gibberellins, cytokinins, siderophores, 1-aminocyclopropane-1-carboxylate (ACC) deaminase, and numerous important nutrients, such as manganese, zinc, and phosphorus, PGPB can convert infertile soils to fertile soils and boost the adaption of a plant to drought stress and other numerous stresses such as light, extreme temperatures, salinity, and diseases. Many plant crops, such as wheat, rice, bean, tomato, and maize (García et al., 2017; Li et al., 2020), can benefit from the application of PGP microorganisms because they increase the availability of nutrients. Overall, plant growth-promoting bacteria as biofertilizers offer a low-cost, environmentally acceptable method of enhancing the development and growth of plants under drought circumstances, making them a crucial tool in facilitating sustainable agriculture (Sarkar and Rakshit, 2020). PGPB are also known to increase the growth and yield of plants as well as agricultural sustainability. This present review concentrated on the impact and mechanisms of action employed by plant growth-promoting bacteria in the mitigation of drought stress in crop plants. It further discussed the challenges encountered in the use of PGPB for plant growth and development as well as in sustainable agriculture.

## Impacts of drought stress on plant growth and health

Drought stress has a significant impact on plant metabolism at all levels, including molecular, morphophysiological, and biochemical (Wang X. et al., 2016). Dehydration produced by moisture stress affects plant metabolic activities such as respiration, chlorophyll content decrease, sugar metabolism, photosynthesis, and nutrient translocation (Dos Reis et al., 2012). Moisture stress also reduces cell water potential and causes the closure of the stomata, which affects the growth and cell elongation. Several crops, including maize, rice, wheat, and barley have been extensively researched under drought stress (Lafitte et al., 2006; Vardharajula et al., 2011). According to Kaur and Asthir (2017), drought stress occurring during the reproductive period might cause blooming to be disrupted, resulting in yield loss.

**FIGURE 1:**
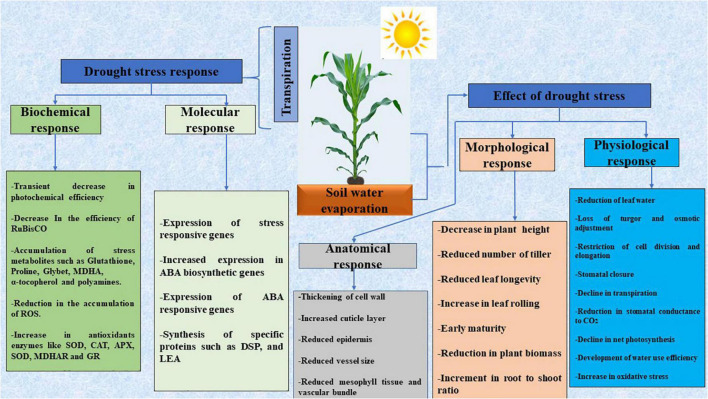
A diagrammatic representation of the responses of plants to drought stress. Adapted from Mamédio et al. (2020). MDHA, Monodehydroascorbate; MDHAR, Monodehydroascorbate reductase; SOD, Superoxide dismutase; APX, Ascorbate peroxidase; POD, Peroxidases; GR, Glutathione reductase; LEA, Late embryo abundant; DSP, Dual-specificity phosphatase.

Reactive oxygen species (ROS) and free radicals, some of which include hydrogen peroxide, superoxide radicals, and hydroxyl radicals are released as a result of drought stress (Figure 1). In plants, greater levels of ROS cause membrane degeneration, lipid peroxidation, nucleic acid, protein, and lipid degradation (Nair et al., 2008). Drought stress causes an increase in the production of ethylene, which is synthesized in response to abiotic stress (Narayanasamy et al., 2020). However, because of the promotion of chlorosis, senescence, and leaf abscission, such an elevated ethylene concentration is harmful to normal plant health and growth. Moisture stress relief is more important than ever to attain food security, given the consequences of moisture deficiency stress and the constantly rising demand for food. As a result, leveraging the potentials of plant and soil-related microorganisms to deal with the negative impacts of drought stress has gotten a lot of attention as a way of boosting crop output (Yang et al., 2009).

## Plant microbes: An intersection between drought stress and plant health

Plant growth-promoting microorganisms (PGPMs) are free-living in soil and are associated with the rhizosphere, rhizoplane, phyllosphere, and endosphere (Babalola et al., 2020; Fadiji and Babalola, 2020a; Fadiji et al., 2020). The roles of endophytic bacteria are less well-reported as rhizospheric as PGPMs in the promotion of plant growth (Bashan and De-Bashan, 2005; Bashan et al., 2014; Fadiji et al., 2021; Sarkar et al., 2022). Plant development is aided by PGPM’s acquisition of nutrients and secretion of phytohormones. In addition, priming of crop plants with PGPM boosts resistance to a variety of stresses, such as salt stress, heavy metals contamination, and drought (Sarkar and Rakshit, 2020; Fadiji et al., 2022; Sarkar et al., 2022). Furthermore, certain PGPMs are biocontrol agents, promoting plant development by inhibiting phytopathogens (Orhan, 2016).

Plant-growth-promoting bacteria also have several action mechanisms, such as siderophore production, ACC deaminase activity, excretion of cell wall degrading enzymes, quorum quenching, and antibiotics which assures induced systemic tolerance (IST) and induced systemic resistance (ISR) in plants to both biotic and abiotic stressors (Olanrewaju et al., 2017; Fadiji and Babalola, 2020a; Akanmu et al., 2021; Sarkar and Rakshit, 2021). *Agrobacterium*, *Bacillus*, *Bradyrhizobium*, *Burkholderia*, *Caulobacter*, *Erwinia*, *Pseudomonas*, and *Rhizobium* are among the well-studied PGPB (De Souza Vandenberghe et al., 2017).

### Plant growth-promoting bacteria

Plant-growth-promoting bacteria are members of the community of microbes associated with plants flourishing in a variety of environments. To increase their growth, these microbes consume organic compounds from the rhizosphere of plants, some of these include sugars (trehalose), and amino acids like glutamine and betaine, among others. Intracellular and extracellular PGPB are the two types (Yadav, 2010). Intracellular PGPB are bacteria that promote plant development but only on the interior of the root surface/rhizoplane root. Likewise, extracellular PGPB boost growth characteristics and the root surface colonization area or the cortex’s intercellular space (Abdelaal et al., 2021). Endophytic growth was initially identified as a very advanced stage of the bacterial lifecycle in 1926 (Hennig, 1940; Fadiji and Babalola, 2020b). After which several endophytes were similarly identified from plant tissues that had been surface-disinfected (Mondal et al., 2020). *Azospirillum*, *Azotobacter*, *Bacillus*, *Erwinia*, *Micrococcus*, *Pseudomonas*, and *Serratia*, are among the extracellular PGPB, whereas *Rhizobia* such as *Mesorhizobium*, *Bradyrhizobium*, and *Allorhizobium*, alongside *Frankia*, are among the intercellular PGPB (Rai et al., 2020).

Endophytic bacteria are symbionts that spend most of their lives within the tissues of the plant without causing harm to their host (Abdelaal et al., 2021) and may be isolated from the interior tissues of the plant (Odoh et al., 2020). Plant development can be aided by PGPB through direct or indirect methods. Increased nutrient availability, such as nitrogen, iron, and phosphorus, alongside increased phytohormone synthesis, is used for the direct enhancement of plant growth (Rastegari et al., 2020). Tolerance to a variety of phytopathogens is developed by an indirect process including antagonistic effects (Glick, 2014). The direct strategy of PGPB includes secretion of siderophores and phytohormones, phosphate solubilization, and nitrogen fixation, all of which induces plant metabolism and improve plant growth (Singh et al., 2020), while indirect strategies include an increment in the activity of defense-related enzymes such as zeatin, chitinase and IAA, ethylene, gibberellic acid (GA3), and abscisic acid which helps in the maintenance of the root system and, as a result, lead to an increment in water uptake and availability of nutrients.

Several studies have found that PGPB often secrete enzymes that degrade phytopathogen cell walls, such as chitinases and proteases (Beneduzi et al., 2012; Fadiji and Babalola, 2020a; Sarkar et al., 2022). Furthermore, the production of antibiotics such as pyoluteorin, phenazines, hydrogen cyanide, phloroglucinols, and pyrrolnitrin, alongside bacteriocins and siderophores, has been shown to aid not only in the inhibition of phytopathogen development (Yadav and Yadav, 2018) but also in the improvement of plant’s tolerance to various stresses (Abdelaal, 2015). It has been proposed that the inoculation of bacteria and other plant growth-promoting organisms (consortia) may also have additive or synergistic effects on plant growth and health (Santoyo et al., 2021).

Drought stress in millet was alleviated by bacterial strains isolated from foxtail millet in a semi-arid agroecosystem that produced EPS and ACC deaminase (Niu et al., 2018). Drought-tolerant bacteria, such as *B. endophyticus*, *B. tequilensis*, and *P. aeruginosa* increased tolerance to drought stress in Arabidopsis seedlings by secreting EPS and phytohormones (Ghosh et al., 2019). Ilyas et al. (2020) also reported that *B. subtilis* and *A. brasilense* produced significant quantities of osmolytes and EPS that increased plant drought tolerance in wheat. The co-inoculation of these microorganisms resulted in greater levels of ABA, proline and EPS synthesis, as well as changes in stress-induced phytohormone levels. However, in response to plants under osmotic stress, germination of seeds, growth of the plant, and seedling vigor index, all increased in response to the inoculation of these microbes (Ilyas et al., 2020).

Drought tolerance in *Medicago truncatula* infected with *Sinorhizobium* sp. was increased by upregulating translation of the jasmonic acid signaling pathway and downregulating ethylene production (Staudinger et al., 2016). Also, when compared to untreated drought-stressed control plants, potato plants treated with *B. subtilis* HAS31 showed greater soluble proteins, total soluble sugars and chlorophyll, as well as higher peroxidase, superoxide dismutase, and catalase enzyme activity under drought stress (Batool et al., 2020).

### The impact of plant-growth-promoting bacteria in ameliorating drought stress

The usefulness of microbial inoculations for promoting plant development exposed to drought stress has been investigated in several studies (Abdelaal et al., 2021; Santoyo et al., 2021). PGPB are well-known for boosting the development of a variety of plants under harsh conditions, including vegetables, grains, and legumes (Sandhya et al., 2010; Abdelaal et al., 2021; Fadiji et al., 2022). PGPB are used to lessen the negative effects of environmental pressures on plant growth and production, through the enhancement of nutrient absorption and increasing tolerance to environmental stress (Ngumbi and Kloepper, 2016). Numerous studies have shown that rhizobacteria can assist to mitigate the harmful effects of different stressors on crop development (Curá et al., 2017). Drought is one of the most damaging conditions affecting plant productivity in semiarid and arid areas (Maheswari et al., 2013). PGPB are useful and beneficial for enhancing plant development exposed to drought stress (Nadeem et al., 2010, 2014), because rhizobacteria may create exopolysaccharides like cellulose and alginate, which are known to aid drought tolerance (Zahir et al., 2009; Glick, 2014). Exopolysaccharides may thus play a crucial role in stress reduction for both microbial and plant populations under drought conditions.

Exopolysaccharides (EPS) are known for the formation of a zone of attachment between soil particles, root systems and bacteria themselves. A few PGPB creates EPS, which may be used as a barrier for protection for the roots and also aid plant development in salinity-stressed conditions (Yang et al., 2016). Biopriming of quinoa seed with *Bacillus* sp. MN54 and *Enterobacter* sp. MN17 and resulted in better plant development under drought environments (400 mM NaCl) (Atouei et al., 2019). Furthermore, *B. subtilis* inaquosorum and *Marinobacter lypoliticus* SM19 were shown to mitigate the effect of salinity and drought stresses in soybean (Miransari and Smith, 2009). Genistein (a nod gene inducer) was reported to increase soybean growth and nodulation, and such effects became greater under high salinity levels over time.

Lipo-chitooligosaccharides are also secreted by PGPB; these compounds are produced by rhizobia and stimulated by flavonoids found in root exudates. *Bradyrhizobium japonicum* 532C inoculated soybean increased growth under salinity and drought stress (Gepstein and Glick, 2013). Trehalose is a non-reducing disaccharide that is present in plants, bacteria, fungi, and insects. It aids plant tolerance to abiotic conditions such as salt and drought stresses. Trehalose is known to be a stable molecule that is highly resistant to acidity and temperatures; it can also ameliorate the effects of drought and salt stresses by avoiding protein aggregation and breakdown of protein, which happens under a variety of stress conditions (Jalili et al., 2009).

The enhancement of plant growth under drought stress may be a result of ACC-deaminase produced by certain strains of PGPB, an enzyme that improves the absorption of some essential nutrients such as K, N, and P, thereby enhancing the growth of plants grown under different environmental conditions (Vaishnav et al., 2016). Application of the PGPB *A. brasilense* and *H. seropedicae* improved drought tolerance in maize (Curá et al., 2017); the positive outcome recorded may be a result of an increment in water use efficiency and enhanced activity of antioxidant enzymes exposed to drought stress.

Additionally, PGPB boosts growth characteristics by generating plant growth hormones such as cytokinins (CKs), gibberellins (GAs), and IAA which increase nitrogen fixation and promote nutrient absorption (Tiwari et al., 2020). Furthermore, due to the buildup of abscisic acid (ABA), PGPB plays an important role in reducing drought stress resistance. PGPB, on the other hand, accumulates osmoprotectants and antioxidants, which might help root development in response to stress (Sharma et al., 2019). The synthesis of IAA by *Azospirillum* species resulted in better root development and increased lateral root formation under drought (Zahir et al., 2009). Under drought stress, inoculating *Lavandula dentate* with *B. thuringiensis* resulted in improved metabolic activity and nutrient absorption in plants (Vurukonda et al., 2016). Inoculation with *Pseudomonas* and *Acinetobacter* species also acclimated grapevine and Arabidopsis plants to drought stress (Armada et al., 2016). In *Platycladus orientalis*, foliar treatment of *Bacillus* stimulates stomatal conductance, increment in ABA, and increases water content under drought conditions (Radhakrishnan et al., 2014).

In another research, *Pseudomonas* inoculation of soybean plants increased the stem height, and fresh weight under drought stress, with similar increases in ABA, and salicylic acid (SA) concentrations, in contrast to the control (Abdelaal et al., 2021). Furthermore, the use of *A. brasilense* and *H. seropedicae* in wheat cultivars resulted in increased drought tolerance, membrane stability, and relative water content (Xu et al., 2018), all of which were linked to a variety of mechanisms such as osmolyte accumulation, ACC-deaminase activity, antioxidant activation and hormonal activity (Barnawal et al., 2017). The buildup of proline and numerous suitable solutes like osmolytes is said to mediate the beneficial effects of PGPB on plants like barley, especially in the enhancement of tolerance to stress (Saikia et al., 2018). Under drought stress, PGPB can directly or indirectly promote ACC deaminase activity, which boosts plant growth (Cakmakci et al., 2007). This method is dependent on PGPB consuming ACC before it is oxidized by plant-produced ACC oxidases. As a result of their ability to reduce ethylene concentrations, PGPB may be a good source of stress tolerance and growth promoters (Santoyo et al., 2016). PGPB were shown to increase plant development in wheat by increasing the activity of ACC deaminase and controlling ethylene levels (Din et al., 2019). Drought-tolerant PGPB which can produce ACC deaminase, inoculated into maize seedlings, enhanced drought tolerances and boosted water and nutrient intake from the soil, resulting in improved plant development (Mohamed et al., 2019).

In a similar report, Bhattacharyya and Jha (2012) found that applying *Pseudomonas* sp. 4MKS8 to maize plants increased agronomic features such as root elongation. Furthermore, inoculation with *Enterobacter cloacae* 2WC2 helped inoculated plants under water stress maintain their water content and develop their root system (Casanovas et al., 2002). Drought stress increased electrolyte leakage and catalase activity in maize genotype TP 30, alongside damage to the membrane, which might be attributable to oxidative stress. In maize plants exposed to drought stress, *Bacillus* spp. treatment resulted in reduced electrolyte leakage, membrane damage, and improved membrane stability as a result of elevated activity of an antioxidant enzyme (Vardharajula et al., 2011). PGPB can reduce the side effects of salt and drought stress by producing ACC deaminase, cytokinin, trehalose, exopolysaccharides, organic compounds, and abscisic acid (Forni et al., 2017). PGPB have been found as a promising way of boosting the yield and growth of drought-stressed crops (Table 1).

**TABLE 1:** Impact of PGPB in the enhancement of drought tolerance in crop plants.

Crop plants	PGPB involved	Impact on plant	References
Pepper	*B. licheniformis* K11	IAA and ACC deaminase produced by the PGPB helps in the mitigation of stress and also in the modulation of the genes and proteins involved in stress response such as sHSP Cadhn, and CaPR-10	Lim and Kim, 2013
Peach	*B. cereus* AR156 *B. subtilis* SM21	Production of ROS scavenging enzymes which often lead to a reduction in lipid peroxidation and boost the protection of plant membranes	Wang et al., 2013
Potato	*B. pumilus* strain DH-11	Enhancement of the efficiency of photosynthesis *via* an increment in ROS production	Gururani et al., 2013
Rice	*P. synxantha* R81 *P. jessenii* R62 *A. nitroguajacolicus* YB3 *B. cereus* BSB38	Activation of antioxidative defense system which results in the alleviation of oxidative stress in crop plants.	Gusain et al., 2015
Pea	*Pseudomonas* sp.	Alteration in the architecture of the root system and ACC deaminase production	Arshad et al., 2008
Sorghum	*Bacillus* sp. K142 and K122	Improvement of plant growth of crops exposed to stress. Such as increment in the content of relative water, shoot length, chlorophyll root dry biomass, and proline content	Grover et al., 2014
Wheat	*B. amyloliquefaciens* 5113	Bacterial priming in the plants reduced reactive oxygen species levels in drought-stressed plants.	Kasim et al., 2013
Rice	*S. yanoikuyae*	Enhancement of antioxidant enzymes, plant growth, and relative water content and in contrast to the control	Arunthavasu et al., 2019
Maize	*B. thuringiensis* HYDGRFB19 *B. licheniformis* HYTAPB18 *P. favisporus* BKB30 *B amyloliquefaciens* HYD-B17	Production of antioxidant enzymes and osmolytes	Daniel, 2019
Green gram	*P. fluorescens* strain Pf1 *B. subtilis* EPB5	Proline content accumulation and antioxidant enzymes for the enhancement of drought tolerance.	Saravanakumar et al., 2011
Sunflower	*A. xylosoxidans* SF2 *B. pumilus* SF3	Increment in phytohormones production	Castillo et al., 2013
Rice	*B. altitudinis* FD48	Proline content accumulation and increased plant biomass.	Narayanasamy et al., 2020
Black gram	*Rhizobium* sp. VRE1	Increased vigor index, germination efficiency, and production of exopolysaccharide.	Raja and Uthandi, 2019; Annadurai et al., 2020
Rice	*B. megaterium*	Alteration in the architecture of the root system for drought stress enhancement.	Vidhyasri et al., 2019
Potato	*A. xylosoxidans Cm4*, *P. oryzihabitans Ep4*, and *V. paradoxus 5C-2*	Modulation of phytohormone levels	Belimov et al., 2015
Black gam and Pea	*O. pseudogrignonense RJ12*, *Pseudomonas* sp. *RJ15*, *B. subtilis RJ46*	Elevated cellular osmolyte and ROS synthesis, higher leaf chlorophyll content, and increased relative water content	Saikia et al., 2018
Wheat and Maize	*Bacillus* sp. (12D6) and *Enterobacter* sp. (16i)	Increased indole-3-acetic acid (IAA) and salicylic acid (SA)	Jochum et al., 2019
Maize	*B. velezensis D3*	Increased vapor, pressure, photosynthesis rate, transpiration rate, stomatal conductance, and water-use efficiency.	Nadeem et al., 2021
Maize	*B. licheniformis*, *B. amyloliquefaciens*, and *B. laterosporus*	Alteration of plant metabolic pathways, including pathways, encoding redox homeostasis, strengthening of the plant cell wall, energy production, membrane remodeling, and osmoregulation.	Nephali et al., 2021
Wheat	*S. maltophilia* and *A. brasilense NO40 B11*	Proline content accumulation and the activities of peroxidase and catalase	Kasim et al., 2021
Maize	*B. cereus* (DS4) and *B. albus* (DS9)	Production of phytohormones and antioxidant enzymes for the enhancement of drought tolerance.	Ashry et al., 2022
Broad bean	*R. leguminosarum biovar viciae* (USDA 2435) and *Pseudomonas putida* (RA MTCC5279)	Increased antioxidant enzyme activities and osmoprotectants	Mansour et al., 2021

## Potential mechanisms used by plant growth-promoting bacteria in mitigating drought stress

**FIGURE 2:**
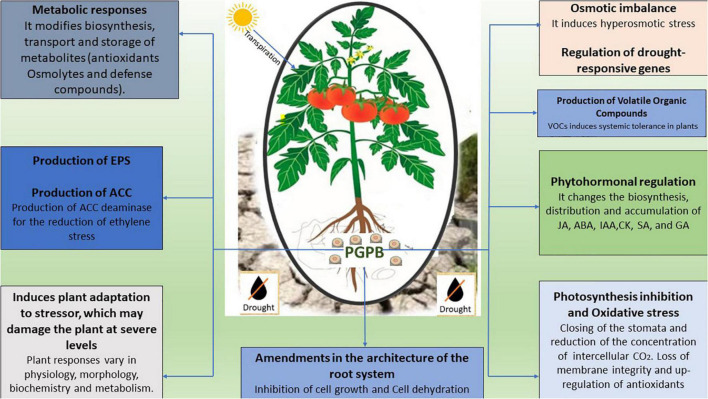
A diagrammatic representation of different potential mechanisms employed by PGPB in the amelioration of drought stress in crops.

Induced systemic tolerance (IST) refers to a microorganism-mediated response to abiotic stress responses of agricultural plants. Drought stress is mitigated by the plant-associated microbiome, which has innate genetic and metabolic characteristics (Gopalakrishnan et al., 2015). Many plant-associated microorganisms from the genera *Azospirillum*, *Bacillus*, *Enterobacter*, *Methylobacterium*, and *Pseudomonas* (Vardharajula et al., 2011; Tiwari et al., 2016; Abdelaal et al., 2021), have been well-studied for their importance in the enhancement of drought tolerance and plant growth improvement. The plausible explanations for plant-associated bacteria-induced tolerance to drought stress are (1) root architecture modification (2) phytohormones production such as cytokinin, gibberellic acid, abscisic acid, and auxins (3) production of ACC deaminase for ethylene stress reduction, (4) production of volatile organic compounds (VOCs) induced systemic tolerance, and (5) exopolysaccharides production (Dimkpa et al., 2009; Vurukonda et al., 2016; Figure 2).

Plant-associated microorganisms also improve stress tolerance in plants by antioxidant production, modulating drought stress-adaptive genes, metabolite and osmolyte synthesis (Jalili et al., 2009). Apart from plants’ capacity to support the colonization of the free-living PGPB population in the soil, the efficiency of PGPB in drought stress tolerance might be connected to interactions between the type of soil and PGPB strains. The function of PGPB-mediated moisture stress resistance is also influenced by the duration and degree of stress, alongside the stage of development of the plant during drought exposure (Ngumbi and Kloepper, 2016). There is a huge number of findings available that explained mechanisms used by PGPB in mitigating the drought stress in crop plants (Table 2).

**TABLE 2:** Mechanisms used by some selected PGPB for the enhancement of plant drought tolerance.

PGPB	Notable mechanisms	References
*Pseudomonas* spp.	Regulation of the pathway involved proline biosynthesis pathway and production of exopolysaccharides (EPS).	Sandhya et al., 2010
*P. chlororaphis* O6	Production of EPS and production of phytohormones and ACC Deaminase	Vurukonda et al., 2016
*A. brasilense* and *H. seropedicae*	Improvement of the water content and cell integrity Improvement of the level of phytohormone production and inducement of the defense-related enzymes and proteins.	Furlan et al., 2017
*B. subtilis*	Improvement of the relative water content, Production of plant growth regulators, e.g., cytokinin	Liu et al., 2013
*B. subtilis* RJ46 and *Pseudomonas* sp. RJ15	Regulation of the level of ethylene Production of ACC deaminases	Saikia et al., 2018
*B. pumilu*	Increment in enzyme activity such as CAT	Gururani et al., 2013
*B. subtilis* (LDR2)	Reduction in the content of ACC Increment in the content of IAA Regulation of the rate of optimal transpiration	Barnawal et al., 2017
*Bacillus* spp.	Increment in the sugar contents Increment in relative water content Decrease in the loss of leaf water	Vardharajula et al., 2011

### Osmolytes concentration in enhancing drought stress tolerance

Plants’ osmolarity concentration has been changed because of adverse environmental circumstances, which has hampered their development and survival. For minimizing dehydration loss caused by moisture stress, osmotic adjustment and the buildup of suitable solutes are required. Sugars, polyamines betaine, proline, quaternary ammonium compounds, amino acids, and dehydrins are some of the compatible solutes that may be implicated in the adjustment of osmolytes. Plants that are stressed can either reduce evaporation or maintain water uptake. One of the vital cellular adaptations in plants exposed to drought stress is osmotic changes, which help in stress tolerance (Farooq et al., 2009). The buildup of osmolytes maintains cellular turgor and reduces the cell water potential while also protecting enzymes and membrane proteins alongside cellular organelles from oxidative damage (Huang et al., 2014). An important example of proteinogenic amino acid is proline which increases during drought stress and is required for key metabolic processes (Huang et al., 2014). Furthermore, by quenching ROS, proline works as a molecular chaperone, lowering lipid peroxidation, altering cytosolic pH, and subcellular structure protection (Zouari et al., 2019). In many plants, proline accumulation during oxidative stress has been linked to drought resistance. Consequently, plant priming with the use of PGPB changes the proline level, which enhances plant drought-tolerant potential. Plants inoculated with PGPB often show an increment in proline levels under drought stress in several trials (Bano et al., 2013; Gusain et al., 2015). Under moisture stress, inoculating maize plants with *Bacillus* sp. boosted proline content (Vardharajula et al., 2011).

Trehalose is a vital osmoprotectant that helps to keep cell signaling and subcellular structures in check (Yang et al., 2009). In a macroarray analysis of nodules of common bean (*Phaseolus vulgaris*) which was coated with *Rhizobium etli*, Suárez et al. (2008) found that the trehalose-6-phosphate synthase gene was overexpressed, indicating an improvement in the production of drought stress-responsive genes in contrast to control. Furthermore, glycine betaine functions as an osmoprotectant, assisting in the stabilization of enzymes and membrane proteins under induced dehydration stress. According to Zhang et al. (2010), coating *Bacillus subtilis* on *Arabidopsis* plants resulted in higher levels of choline, betaine, and glycine than controls. The synthesis of essential metabolites among which are polyamines (putrescine, cadaverine, and spermidine) alongside amino acids boosted the plant’s osmotic tolerance to drought stress, in addition to transcriptional control and cell differentiation. During oxidative stress, Arabidopsis coated with Spd-producing *B. megaterium* BOFC15 showed an increment in the production of polyamines at the cellular level.

The host plants coated with bacteria, often possess a stronger root system with more lateral roots, and an increment in main root length helps the plant in enhancing plant drought stress better than control plants (Zhou et al., 2016). As a result, plants with higher levels of amino acid and soluble sugars are thought to be better able to withstand drought. Under drought stress, Yooyongwech et al. (2017) found a greater amount of soluble sugar content in sweet potatoes colonized with mycorrhiza. Endophytic fungus *Trichoderma hamatum* strain DIS 219b colonized in cocoa seedlings revealed a delay in the physiological changes generated by moisture stress as reported by Bae et al. (2009).

### Production of antioxidants in enhancing drought stress tolerance

Under optimal conditions, the formation of reactive oxygen species (ROS) as a metabolic product in plants is negligible. Skewness is triggered by an increase in photorespiration-induced detoxification and overproduction of ROS, and the disrupted system of photosynthesis is a critical change that occurs under drought stress. According to Cruz De Carvalho (2008), increased formation of a wide range of reactive oxygen species, including hydroxyl radical, superoxide radical, singlet oxygen, and hydrogen peroxide, is one of the probable repercussions of moisture stress.

By causing oxidative stress to a macromolecule, proteins, alongside similar lipids, ROS can disrupt normal plant metabolic operations and even cause the death of cells (Farooq et al., 2009; Hasanuzzaman et al., 2013). Although, a rise in ROS causes oxidative stress, a lower amount of ROS is required by the plants for signaling occurrences that will activate defense mechanisms. To control signaling events and oxidative stress in plants, it is critical to manage reactive oxygen species through controlled regulation of reactive oxygen species generation and quenching. PGPB priming of plants often changes the level of antioxidants, which is an efficient Microbe-induced systemic tolerance (MIST) mechanism in enhancing tolerance to drought, according to several reported studies (Cohen et al., 2015; Ortiz et al., 2015).

Under moisture stress, *Solanum tuberosum* plants primed with plant growth-promoting rhizobacteria (PGPR) showed an improvement in numerous enzymes scavenging the reactive oxygen species (Gururani et al., 2013). Furthermore, under moisture stress, catalase activity was 1.8 times greater than the level observed in the control plants. Green gram infected with *B. subtilis* EPB and *P. fluorescens* Pf1 showed an improvement in the activity of CAT (Saravanakumar et al., 2011). Most plants, such as maize, wheat, and rice, have shown a link between drought resistance and the production of ROS (Vardharajula et al., 2011; Kasim et al., 2013; Gusain et al., 2015).

Seed coating with *B. methylotrophicus* and RABA6 and *B. altitudinis* FD48 in rice enhanced the production of enzymes capable of quenching ROS which aids tolerance to drought stress (Narayanasamy et al., 2020). Eleusine seedlings injected with *R. intraradices* demonstrated enhanced ascorbate, glutathione and flavonoid under drought stress, according to recent findings (Narayanasamy et al., 2020). As a result, plants primed with specific PGPR produce more ROS scavenging enzymes, which reduces ROS overproduction and confers drought resistance.

### Phytohormones modulation to enhance plant tolerance to drought stress

Phytohormones are hormones generated in minute amounts in plant tissues that affect plant development and growth, such as fruit ripening, lateral root growth, blooming, bud initiation, and so on. Ethylene, abscisic acid, cytokinins, indole acetic acid, and gibberellins are plant hormones that assist plants to survive in stressful environments. PGPB can also secrete phytohormones, which drive plant development and aid plant survival in hostile environments. These findings imply that bacterial hormone production and their capacity to activate endogenous hormone levels in plants are important in increasing tolerance (Cassán et al., 2001).

#### Auxins

Auxins, such as IAA, are responsible for apical dominance, cell division, root and shoot growth direction, and later root development among other things (Glick, 1995). IAA can be produced by about 80% of PGPR (Olanrewaju et al., 2017). Earlier studies have proposed that PGPMs can assist plants in managing abiotic stress by supplying IAA, hence helping crop development and growth. Under drought stress, clover plants coated with PGPB’s *B. megaterium* and *P. putida* increased shoot and root biomass, which is associated with increased IAA production (Marulanda et al., 2010).

Furthermore, *Arabidopsis* injected with *P. brassicacearum* STM196 have longer roots, and the architecture of root system modification confers drought resistance (Bresson et al., 2013). The results suggested that IAA may help mitigate bacterial-caused drought to some extent. Inoculation of tomato and wheat seedlings with IAA-producing *Azospirillum* resulted in increased formation of lateral and root development, resulting in increased water and nutrient absorption under drought stress (Vurukonda et al., 2016). In earlier research, Ambreetha et al. (2018) claimed that *B. altitudinis* FD48 inoculation altered the root system architecture in rice, and this was established by examining the mode of expression of genes encoding defense responsive in the formation of primary root (OsIAA4 and OsIAA1) and lateral root (OsIAA13 and OsIAA11). The results showed that inoculating rice with FD48 alters the endogenous level of IAA *via* regulating the genes encoding auxin expression, modulating the architecture of the root system and ensuring the survival of the plant exposed to moisture stress.

#### Abscisic acid

Abscisic acid (ABA) is a critical hormone that performs a key function in a plant’s physiology and is required for tolerance to most abiotic stresses (Cohen et al., 2015). Plant-associated bacteria secrete high levels of abscisic acid which can help plants withstand moisture stress. Under water-stressed situations, increased levels of abscisic acid in plant tissues led to physiological changes which aided plant development (Farooq et al., 2009). Under osmotic stress, abscisic acid activates drought stress-adoptive genes and modulates cell signaling, resulting in the elicitation of greater resistance responses.

Abscisic acid is an antitranspirant that stimulates the closure of the stomatal in addition to signaling (Egamberdieva et al., 2017). Furthermore, in a stressful environment, abscisic acid alters the root system and develops deeper roots, as well as other modifications in auxiliary roots that obstruct water for optimal plant growth and the acquisition of nutrients. Furthermore, ABA preserves the root and shoot hydraulic conductivity to properly use water from the environment, resulting in turgor potential retention in plant tissues. The buildup of abscisic acid in Arabidopsis coated with *P. brassicacearum* strain STM196 was discovered to modify drought response, leading to a lower rate of leaf transpiration. *Bacillus* sp. primed in lettuce produced more ABA than fake plants, according to Vurukonda et al. (2016), resulting in increased moisture stress tolerance. They also found a link between ABA levels and drought tolerance. Similarly, Arabidopsis plants treated with *A. brasilense* Sp245 enhanced their resistance to drought stress by accumulating more ABA (Cohen et al., 2008).

Many studies have been proposed to understand the methods through which ABA might increase drought resistance. One hypothesis is that ABA improves drought tolerance by controlling the hydraulic conductivity of the root and leaf transpiration (Aroca et al., 2008). A similar idea claims that abscisic acid helps in the mitigation of moisture stress by aquaporin regulation (Zhou et al., 2012). It is clear from all the theories that abscisic acid plays an important function in plant development and resistance to moisture stress.

#### Gibberellins

Gibberellins are notable hormones of plants that govern a variety of physiological activities in plants at various phases of development, including blooming, seed germination, senescence, elongation of the stem, and fruiting (Kang et al., 2014a). Numerous studies have suggested that priming GA-producing bacteria might assist host plants to adapt to moisture stress. Drought stress is mitigated by GA generating *Azospirillum lipoferum* stimulated in maize plants, according to a study carried out by Kaushal and Wani (2016). In addition, *P. putida* H-2-3 inoculation is known to induce drought tolerance and produce gibberellins in maize plants. Exogenous treatment of GA, on the other hand, improves root characteristics (such as root tips, surface length, and area), resulting in enhanced nutrient absorption and thereby modifying the functions of plants in stressful conditions (Vacheron et al., 2015).

#### Cytokinins

Photosynthetic activity, plant cell division, and the closure and opening of the stomata under drought are all greatly influenced by cytokinins. Aside from plants, a wide spectrum of soil bacteria and PGPB may produce cytokinins. Plants coated with PGPB also revealed a possible influence of cytokinins on tolerance to drought stress (Arkhipova et al., 2007; Liu et al., 2013; Hai et al., 2020; Abdelaal et al., 2021).

#### 1-aminocyclopropane-1-carboxylate deaminase enhances tolerance to drought stress

Ethylene is a common hormone that serves as an important oscillator for the development and growth of plants alongside serves as a key component in the regulation of plants. Abiotic stress biological signals coordinate ethylene biosynthesis (Hardoim et al., 2008). Upon exposure to environmental challenges, plants are known to generate more ethylene (Abdelaal et al., 2021). Increased ethylene levels, on the other hand, cause chlorosis, senescence, and leaf abscission, which are all harmful to proper plant development and growth. Plant-associated bacteria generated ACC, which decreased the impact of ethylene on stressed plants and improved plant health. S-adenosyl methionine (S-Adomet) is transformed into ACC, which is widely known ethylene precursor in the production of ethylene.

Endogenous ethylene in plants often leads to lower plant growth in stressful situations. The regulation of ACC is a critical mechanism through which the PGPB tries for favorable impacts on plant development and growth in drought-stressed conditions (Saleem et al., 2007). ACC deaminase, a rhizobacteria enzyme that cleaves ACC into a-ketobutyrate and ammonia, lowered the quantity of ACC, and hence the quantity of ethylene, and thus the harmful impact on plants at greater ethylene concentrations, was abolished (Glick, 2014). As a result, it aided plant development by reducing drought stress.

### Production of exopolysaccharides in enhancing drought stress tolerance

Bacterial EPS has been studied extensively in the rhizosphere for its function in moisture retention (Ali et al., 2014). Slime materials and capsular EPS are released into the soil in two forms: slime materials and capsular EPS which may be absorbed through the clay surfaces *via* cation bridges, the Van der Wals force, hydrogen bonding, and anion absorption process provides a protective barrier surrounding soil aggregates (Sandhya et al., 2009). Furthermore, alginate, a tiny polysaccharide existing as a biofilm, helps to maintain a hydrated microenvironment by water retention and drying at a slower pace than the surrounding environment, thus protecting plant roots and microorganisms from desiccation (Sandhya et al., 2009). According to Selvakumar et al. (2012), bacteria-produced EPS increases soil impermeability by enhancing the aggregation of soil and maintaining increased water potential over the root area. As a result, the absorption of nutrients increases, which improves the growth of the plant and resistance to moisture stress.

Plants primed with bacteria capable of producing EPS show enhanced resilience to moisture stress (Ojuederie et al., 2019). When subjected to moisture stress, the seedlings of sunflower injected with *Pseudomonas* sp. GAP-P45 improved the survival of the plants exposed to stress by increasing plant biomass and the RAS/RT ratio. The bacteria were successfully colonized in root-adhering rhizoplane and soil, resulting in increased soil aggregate stability (Sandhya et al., 2009). More RAS aggregation allows for increased water and nutrient absorption from the rhizosphere, allowing plants to survive and flourish in the face of induced drought stress (Sandhya et al., 2009).

### Amendment in the architecture of the root system

The roots of the plant are an essential tissue for the growth of the plant because they help with the absorption and transportation of nutrient water, anchoring, and symbiotic interaction with beneficial microorganisms in the soil, all of which contribute to increased plant health and growth (Hodge et al., 2009; Abdelaal et al., 2021). In drought-stressed plants, the architecture of the root system is the most important characteristic. The structure of the root system, the geographical dissemination of secondary and primary roots, variations in the root number, and root diameter are all included in the root system. Root flexibility in the response of plans to water scarcity is a valuable tool for dealing with moisture stress (Ngumbi and Kloepper, 2016).

The architecture of the root is altered by PGPB-mediated alterations in root development and flexibility. Some of the bacteria-associated changes in the structure of the root are intended to increase the area of the root surface and, as a result, nutrient and water uptake, which has a considerable impact on drought resistance (Ngumbi and Kloepper, 2016; Abdelaal et al., 2021). Under drought stress, maize seedlings primed with *A. faecalis* AF3. Ambreetha et al. (2018) stated that *B. altitudinis* FD48 inoculation altered the architecture of the root system in rice, and this was later corroborated by the pattern of expression of defense-responsive genes encoding the main development of the root (OsIAA4, OsIAA1) and the formation of lateral root (OsIAA13, OsIAA11).

These findings show that inoculating rice with FD48 alters endogenous IAA levels and auxin-responsive genes, thereby resulting in changes to the root system architecture. PGPB-mediated root architecture modification is important for increasing rice yield under extreme environmental circumstances (Paez-Garcia et al., 2015). Many studies have shown that modifying rice root system features can increase yield in stressful settings (Zou et al., 2015). The PGPR produces signaling molecules that naturally change the root system architecture of related crops (Phillips et al., 2004; Ojuederie et al., 2019).

Most PGPB such as *B. megaterium*, and *Pseudomonas* sp. have been widely reported in the modification of the Arabidopsis root architecture (Zamioudis et al., 2013). The development of lateral roots in *B. megaterium* primed *Phaseolus vulgaris* roots was boosted (Lopez-Bucio et al., 2007). As a result, PGPB caused changes in the flexibility of cell membranes of the root, known as the key reactions in the area for increased moisture stress tolerance (Vurukonda et al., 2016). In addition, computer-based analysis and imaging techniques are utilized to compare the root architectural characteristics of the plants, in addition to morphological perception and observation of the root system. Another accurate program used to comprehend the architecture of rice roots is WinRHIZO, GiA Roots, and 3D root imaging (Ambreetha et al., 2018). Under *in vitro* settings, volatile chemicals released by *B. pumilus* T4 and *E. cloacae* JM22 enhance overall biomass and induce adventitious root development (Delaplace et al., 2015).

### Emission of microbial volatile organic compounds to mitigate drought stress

Rhizosphere-dwelling and plant-associated microorganisms can assist the plant in dealing with drought stress through a variety of methods (Ojuederie et al., 2019; Ahluwalia et al., 2021). Recent research has shown that insoluble metabolites produced by rhizospheric microbiomes as microbial volatile organic compounds (mVOC) improve plant drought tolerance. According to Narayanasamy et al. (2020), volatile compounds are interesting candidates for stress reduction during plant development and growth. Wheat seedlings treated with *B. thuringiensis* AZP2 volatiles, for example, showed a fivefold improvement in survival under drought, owing to a large improvement in plant biomass, which enhances photosynthetic rate (Narayanasamy et al., 2020).

Some mVOCs emitted by *B. thuringiensis*, such as geranyl, b-pinene, and benzaldehyde compounds, increase tolerance to drought in wheat. Cho et al. (2008) revealed that microbial volatile organic compound 2R,3R-butanediol generated by *P. chlororaphis* O6 assists in promoting tolerance to drought stress in *Arabidopsis* by the closure of the stomatal. This points to the mVOC’s key significance in tolerance to drought stress and pathways of hormone signaling. Recent research has revealed that 2,3-butanediol stimulates the synthesis of nitric oxide (NO) in plants, which improves the survival of the plant under drought, suggesting that NO signaling plays a vital role in improving tolerance to drought (Cho et al., 2013). *B. subtilis* GB03 also generated VOCs, such as 2,3-butanediol, which is important in drought relief (Zhang et al., 2010). Additionally, Arabidopsis exposed to *B. subtilis* GB03 volatile organic compound**s** had greater levels of glycine betaine and choline accumulation, as well as increased levels of phosphoethanolamine *N*-methyltransferase (PEAMT) transcripts, which are required for glycine betaine and choline production (Mou et al., 2002; Abdelaal et al., 2021).

Many PGP bacterial strains, including *Pseudomonas aeruginosa* Pa2, create exopolysaccharides that assist in maintaining soil moisture content, improving plant drought resistance (Naseem and Bano, 2014). Some mVOCs, such as acetic acid generated by bacteria, can promote the production of biofilms, with EPS being the most important element (Chen et al., 2015). As a result, specific microbial volatile organic compound-mediated exopolysaccharide synthesis in rhizobacteria may affect tolerance to drought indirectly in plants. Inoculation of wheat with *O. pseudogregnonense* IP8 and *B. safensis* W10 resulted in the formation of high amounts of antioxidants and metabolites, which play an important function in the mitigation of drought stress (Chakraborty et al., 2013). PGPR primed potatoes also increased the accumulation of proline and ROS scavenging enzymes and gene expression, indicating improved resistance to abiotic conditions such as salt, the toxicity of heavy metals and drought (Gururani et al., 2013).

### Regulation of drought-responsive genes

Studies involving the expression of a gene are a useful technique for determining and comparing plant responses to their surroundings. The transcriptome research examines the expression of a cell’s whole collection of transcripts (mRNA) at a normal developmental stage or exposed to various environmental circumstances (Vurukonda et al., 2016). Plants colonized by microbes have distinct gene expression patterns than non-colonized plants, resulting in increased tolerance to drought. Comparison-based transcriptomic profiling aids in the identification of diverse groups of gene transcripts responsible for changes in two physiologically distinct expressions under different situations (Bräutigam and Gowik, 2010; Fadiji and Babalola, 2020c). Therefore, some of the most important tools in investigating plant-microbe interactions are transcriptome profiling from microarray and mRNA sequencing (Wang W. et al., 2016).

The physiological function of PGPB was used to assess gene regulation impacted by moisture stress, according to Mohammadipanah and Zamanzadeh (2019). In *Arabidopsis* plants infected with *B. polymyxa* B2, the favorable impact of plant growth-promoting bacteria on improving plant stress tolerance at the transcriptomic level was investigated (Yang et al., 2009; Vurukonda et al., 2016; Abdelaal et al., 2021). These results show that the inoculated plants produce the drought-adaptive gene early response to ABA-regulatory gene *RAB18* (LEA) and dehydration 15 (*ERD15*), but uninoculated sham plants do not.

Similarly, wheat plants infected with *A. brasilense* NO40 and *B. amyloliquefaciens* 5113 demonstrated enhanced drought tolerance as measured using real-time qPCR, with activation of genes that are stress-responsive (*HSP17*, *SAMS*1, and *APX*1) in the leaves of wheat. Microarray investigation of *A. thaliana* primed with *P. chlororaphis* O6 demonstrated downregulation of the signaling gene for responses to drought as compared to plants without being colonized by bacteria, according to Vurukonda et al. (2016).

In addition, the bacterium-primed plants caused activation of the genes signaling jasmonic acid *VSP1* and *PDF-1.2*, the salicylic acid regulatory gene *PR1*, and the ethylene-responsive factor HEL. The impact of stress-responsive genes such as OsWRKY11, *OsDREB*1A, *OsDIL*, *OsGADPH*, *OsAP*37, and *OsNAC*6 was upregulated in rice plants primed with co-inoculation of two PGPR strains, namely *B. amyloliquefaciens* BK7 and *B. laterosporus* B4, indicating the microbe-induced systemic drought tolerance (Kakar et al., 2016). Similarly, qPCR analysis of *P. fluorescens* strain Pf1 primed rice revealed upregulation of six drought-responsive genes during drought stress, including *COX1*, *PKDP*, *Hsp20*, *AP2-EREBP*, *COC1*, and *bZIP1*. Rice plants treated with *T. harzianum* elevated drought-related genes such as aquaporin, malondialdehyde, and dehydrin, as well as physiological indices, according to Pandey et al. (2016). It also boosts phytohormones, antioxidant enzymes, and defense proteins, all of which contribute to drought resistance and endurance. In *P. trifoliata* infected with Arbuscular mycorrhizal fungi (*F. mosseae*). Liu et al. (2018) found that root genes *PtYUC*3 and *PtYUC*8 linked to IAA production were upregulated under drought stress and normal conditions. During water deficiency stress, the transcriptional levels of *PtLAX*2 and *PtABCB*19 (root auxin species inflow carriers) increased, whereas the expression levels of *PtPIN*3 and *PtPIN*1 (root auxin efflux carriers) decreased. These findings highlighted the usefulness of transcriptome profiling as a tool for studying the functional genomes of plants, and it contains multiple potential genes as a natural indicator of drought resistance.

## Major constraints in the applications of plant-growth-promoting bacteria-induced drought tolerance

Drought stress is a major danger to agriculture and the production of food in the long run. It produces a buildup of ROS, which results in oxidative stress in plants. Furthermore, it causes a decrease in agricultural output and a loss of income for farmers. It is critical to have a cheaper method of increasing agricultural output under abiotic stress if the ever-increasing population of humans is to be fed satisfactorily. The loss of farmland, the absence of regulated water sources, and the long-term impacts of climate change have the potential to be disastrous to food production (Kasim et al., 2013; Abdelaal et al., 2021).

Various ways to reduce the impact of drought on plants have been developed, with the most concentrated being the production of drought-resistant cultivars, which includes current biotechnology technologies (Ashraf, 2010). However, because the processes of enhancing tolerance to drought stress are ambiguous, the introduction of novel drought-resistant cultivars is critical, as genetically modified crops are widely rejected in most countries of the world. Plant-associated microorganisms are an underappreciated source of drought resistance in agricultural plants. Plant development and growth are ensured by the widespread use of microbial inoculants, although the research is restricted to controlled circumstances. In natural settings, the results obtained from earlier research do not achieve a suitable level of accuracy and efficacy for commercialization on a worldwide scale. This might be owing to the intricate interactions being defined by soil physicochemical characteristics and microbial populations (Nadeem et al., 2014).

Plant-associated microorganisms can colonize the above plant’s tissues or roots, enhancing development and abiotic stress tolerance through biological modulation of phytopathogens. One of the most difficult challenges in this science is identifying distinct strains of the beneficial microbiome as well as their likely functional roles. It’s crucial to figure out how the notable microbiome works and what function it plays in agricultural sustainability (Pujar et al., 2017). The associative microbiome’s relationship with the plant may be inherently unstable. Some results acquired *in vitro* are not always expected to be comparable to those achieved in the field. The plant-associated bacteria’s poor coordination might be owing to environmental restrictions that affect plant development and growth. Soil qualities, climatic circumstances, dynamics, and the activity of the microbial population in the soil are examples of such restrictions.

## Conclusion and future perspectives

Crops treated with microbial inoculants employ a variety of methods to battle drought stress, resulting in higher crop yields. Using PGPB’s capacity to promote plant development and defense against drought stress is a cost-effective, environmentally friendly, and promising way to reduce the impacts of drought stress on crops. The PGPB functional properties mentioned in this review are also fundamental methods by which PGPB enhance plant development and growth. Plant development under drought stress is also affected by PGPB interactions, which enhance antioxidant enzymes and synthesis of osmolytes, as well as the expression of drought-adaptive genes. As a result, multifunctional PGPB are crucial for long-term food security and agricultural production, especially in harsh environments. Whatever functional qualities PGPB has in drought stress mitigation, the primary problems are colonization, competence, survival in non-native soils, and exerting the required advantages under field settings. It is critical to investigate the mechanism by which PGPB impose their drought-tolerant effects on plants, as well as the impacts of numerous environmental factors, alongside other microbial interactions, to achieve the best growth-promoting relationship between seedlings and beneficial microorganisms. As a result, developing competent strains for field circumstances or a specific location is a key task. We are only at the beginning of our knowledge of the processes of PGPB in plants, as evidenced by the fast growth and prognosis of microbial-induced drought stress resistance in plants.

Nonetheless, recent development in the sector indicates that future study has enormous prospects to bring new insights into food production and sustainability. Newer formulations, such as nano encapsulations, might be researched to assure the efficiency of bioinoculants in field circumstances for better drought tolerance, colonization and distribution of beneficial microbes on host plants. Furthermore, PGPB affects not only the plants but also the soil qualities when there is a drought. As a result, more molecular studies into plant-microbe interactions are needed in the future to understand the routes employed by rhizospheric microorganisms in induced systemic tolerance and rhizosphere engineering under drought stress. To reap these benefits, future studies must focus on identifying the optimum sort of potential strain, adequate mechanism of delivery, and field evaluation for long-term crop and food production.

## Author contributions

AF and OB conceived the ideas, collected the data, and developed the manuscript. GS and AY provided professional input and critiqued the work. All authors approved the submitted version.
